# Supplementary material for: Are Housing Prices Associated with Food Consumption?
Source: Int J Environ Res Public Health. 2020 May 30;17(11):3882. doi: 10.3390/ijerph17113882 (PMC7312069; doi:10.3390/ijerph17113882)
Supplement: Supplementary file 1 [file ijerph-17-03882-s001.pdf]

Table S1. Age differential marginal effects of housing value on four categories of food consumption, stratified by home ownership status.

	Renters				Homeowners			
Age Group	All Veg	Fruit	Legume	Juice	All Veg	Fruit	Legume	Juice
				# servings per week				
18–24	−0.043	−0.157	0.015	−0.064	0.020	−0.034	−0.021	−0.039*
	(0.114)	(0.086)	(0.034)	(0.035)	(0.050)	(0.039)	(0.015)	(0.017)
25–34	−0.030	−0.120	−0.036	−0.051	0.012	−0.055	−0.009	−0.006
	(0.095)	(0.077)	(0.030)	(0.032)	(0.043)	(0.034)	(0.013)	(0.014)
35–44	−0.007	−0.058	0.030	−0.049	−0.011	−0.038	−0.020	−0.008
	(0.103)	(0.074)	(0.033)	(0.030)	(0.040)	(0.030)	(0.014)	(0.011)
45–55	−0.064	−0.040	−0.009	−0.035	0.013	−0.037	−0.024	−0.006
	(0.109)	(0.085)	(0.034)	(0.032)	(0.039)	(0.029)	(0.014)	(0.011)
55–64	0.012	−0.119	−0.024	−0.047	0.010	−0.035	−0.018	−0.008
	(0.104)	(0.080)	(0.034)	(0.032)	(0.039)	(0.030)	(0.014)	(0.011)
65+	−0.084	−0.232**	−0.036	−0.058	0.023	−0.055	−0.027*	−0.014
	(0.106)	(0.083)	(0.031)	(0.036)	(0.038)	(0.031)	(0.013)	(0.012)
				≥2 servings per day				
18–24	−0.52	−0.15	0.08	0.00	0.13	−0.25	−0.35*	−0.32
	(0.47)	(0.44)	(0.15)	(0.33)	(0.23)	(0.23)	(0.16)	(0.18)
25–34	−0.09	0.05	−0.03	0.00	−0.12	−0.46*	−0.09	−0.09
	(0.43)	(0.38)	(0.15)	(0.33)	(0.19)	(0.19)	(0.05)	(0.13)
35–44	−0.13	0.17	0.08	−0.00	−0.25	−0.26	−0.24*	−0.08
	(0.46)	(0.40)	(0.17)	(0.28)	(0.18)	(0.16)	(0.09)	(0.10)
45–55	0.07	0.17	0.05	0.12	−0.11	−0.26	−0.16*	−0.03
	(0.46)	(0.42)	(0.18)	(0.28)	(0.17)	(0.16)	(0.07)	(0.10)
55–64	−0.00	−0.02	−0.04	−0.03	−0.12	−0.29	−0.14*	−0.08
	(0.46)	(0.42)	(0.19)	(0.31)	(0.18)	(0.17)	(0.07)	(0.08)
65+	0.33	−0.06	0.08	0.20	−0.05	−0.34*	−0.16**	−0.06
	(0.48)	(0.42)	(0.14)	(0.33)	(0.17)	(0.16)	(0.06)	(0.08)

* $p < 0.05$, ** $p < 0.01$, *** $p < 0.001$. [1.]Change in times per week a food category is consumed attributable to a $100 increase metropolitan/micropolitan (MMSA) level home rental prices (for renters) or a $10,000 increase in home values (for homeowners), conditional on age group. [2.]Change in the probability of consuming a food category ≥2 times per day attributable to a $100 increase metropolitan/micropolitan (MMSA) level home rental prices (for renters) or a $10,000 increase in home values (for homeowners), conditional on age group.

Table S2. Race differential marginal effects of housing value on four categories of food consumption, stratified by home ownership status.

	Renters				Homeowners			
**Race Group**	**All Veg**	**Fruits**	**Legume**	**Juice**	**All Veg**	**Fruits**	**Legume**	**Juice**
				**Frequency per week**				
NH−White	−0.009	−0.071	−0.003	−0.053*	0.017	−0.050	−0.015	−0.009
	(0.094)	(0.068)	(0.025)	(0.026)	(0.038)	(0.029)	(0.012)	(0.010)
NH−Black	0.006	−0.137	0.026	−0.046	−0.006	−0.085*	−0.010	−0.015
	(0.097)	(0.088)	(0.029)	(0.046)	(0.043)	(0.034)	(0.013)	(0.019)
Hispanic	−0.224	−0.303***	−0.059	−0.066	−0.041	−0.080*	−0.041*	−0.012
	(0.122)	(0.088)	(0.034)	(0.038)	(0.045)	(0.034)	(0.016)	(0.013)
NH−Other	−0.251	−0.267*	−0.001	−0.073	−0.068	−0.109*	−0.021	−0.013
	(0.158)	(0.124)	(0.037)	(0.074)	(0.062)	(0.049)	(0.017)	(0.025)
NH−Multi−Race	−0.096	−0.166	−0.060	−0.016	0.033	−0.021	−0.031	−0.020
	(0.118)	(0.101)	(0.051)	(0.034)	(0.043)	(0.038)	(0.020)	(0.015)
				**≥2 servings per day**				
NH−White	0.00	0.23	0.06	−0.01	−0.07	−0.35*	−0.08*	−0.03
	(0.41)	(0.35)	(0.08)	(0.22)	(0.17)	(0.15)	(0.04)	(0.07)
NH−Black	0.11	−0.17	0.09	0.36	−0.16	−0.65***	−0.14*	−0.20
	(0.44)	(0.45)	(0.15)	(0.51)	(0.19)	(0.19)	(0.06)	(0.16)
Hispanic	−0.77	−1.02*	−0.11	0.19	−0.26	−0.40*	−0.25*	−0.06
	(0.62)	(0.49)	(0.21)	(0.31)	(0.21)	(0.19)	(0.11)	(0.09)
NH−Other	−1.97*	−1.08	0.10	−0.17	−0.45	−0.69*	−0.17	−0.07
	(0.85)	(0.76)	(0.12)	(0.80)	(0.34)	(0.29)	(0.11)	(0.25)
NH−Multi−Race	−0.13	0.09	0.07	0.20	−0.12	−0.20	−0.34**	−0.22
	(0.48)	(0.45)	(0.30)	(0.30)	(0.20)	(0.20)	(0.13)	(0.14)

* $p < 0.05$, ** $p < 0.01$, *** $p < 0.001$. NH = non−Hispanic. [1.]Change in times per week a food category is consumed attributable to a $100 increase metropolitan/micropolitan (MMSA) level home rental prices (for renters) or a $10,000 increase in home values (for homeowners), conditional on race/ethnicity group. [2.]Change in the probability of consuming a food category ≥2 times per day attributable to a $100 increase metropolitan/micropolitan (MMSA) level home rental prices (for renters) or a $10,000 increase in home values (for homeowners), conditional on race/ethnicity group.

Table S3. Education differential marginal effects of housing value on four categories of food consumption, stratified by home ownership status.

	Renters				Homeowners			
**Education Group**	**All Veg**	**Fruits**	**Legume**	**Juice**	**All Veg**	**Fruits**	**Legume**	**Juice**
				**Frequency per week**				
< HS	0.005	−0.049	0.028	−0.034	0.025	−0.002	−0.007	−0.002
	(0.103)	(0.079)	(0.037)	(0.033)	(0.042)	(0.037)	(0.016)	(0.016)
HS Grad	0.012	−0.096	0.006	−0.036	0.033	−0.039	−0.019	−0.006
	(0.095)	(0.076)	(0.032)	(0.032)	(0.036)	(0.028)	(0.012)	(0.011)
Some college	−0.052	−0.108	−0.014	−0.052	0.006	−0.045	−0.023	−0.013
	(0.095)	(0.071)	(0.027)	(0.032)	(0.039)	(0.030)	(0.013)	(0.012)
College Grad	−0.089	−0.210**	−0.042	−0.069*	−0.011	−0.069*	−0.024	−0.014
	(0.101)	(0.081)	(0.032)	(0.029)	(0.042)	(0.033)	(0.014)	(0.011)
				**≥2 servings per day**				
< HS	−0.10	0.40	0.08	0.10	−0.10	−0.15	−0.20	−0.13
	(0.47)	(0.41)	(0.23)	(0.33)	(0.20)	(0.19)	(0.11)	(0.14)
HS Grad	−0.02	0.06	−0.04	−0.06	0.01	−0.27	−0.14**	−0.05
	(0.41)	(0.36)	(0.14)	(0.30)	(0.16)	(0.15)	(0.05)	(0.10)
Some college	−0.10	−0.07	0.06	0.13	−0.13	−0.31	−0.18**	−0.08
	(0.42)	(0.37)	(0.11)	(0.31)	(0.18)	(0.16)	(0.06)	(0.10)
College Grad	−0.22	−0.21	0.02	−0.07	−0.19	−0.43*	−0.16**	−0.08
	(0.47)	(0.41)	(0.15)	(0.23)	(0.19)	(0.18)	(0.06)	(0.08)

* $p < 0.05$, ** $p < 0.01$, *** $p < 0.001$. HS = high school. [1.]Change in times per week a food category is consumed attributable to a $100 increase metropolitan/micropolitan (MMSA) level home rental prices (for renters) or a $10,000 increase in home values (for homeowners), conditional on educational attainment. [2.]Change in the probability of consuming a food category ≥2 times per day attributable to a $100 increase metropolitan/micropolitan (MMSA) level home rental prices (for renters) or a $10,000 increase in home values (for homeowners), conditional on educational attainment.
